# Oral Narrative Intervention by Tele-Practice in a Case with Developmental Language Disorder

**DOI:** 10.3390/children8111052

**Published:** 2021-11-14

**Authors:** Irina Iuliu, Verónica Martínez

**Affiliations:** Department of Psychology, University of Oviedo, 33003 Oviedo, Spain; UO265359@uniovi.es

**Keywords:** Developmental Language Disorder, speech and language intervention, narrative skill, tele-practice

## Abstract

Background: A narrative requires the integration and management of linguistic and cognitive skills. It has been observed that children with Developmental Language Disorder (DLD) have difficulties in narrating stories. This research proposes an intervention in a case of a child 9 years and 2 months old with DLD, with the aim of improving his oral narrative skills through a retelling task via telepractice. Methods: In the evaluation, standardized tests have been used and a ‘remembering a story’ task, with a story titled *The Lost Backpack*, elaborated by one of the authors. Narratives were elicited in two sessions, and were transcribed, coded, and analysed using the Child Language Data Exchange System CHILDES Project tool. The participant received a total of 10 sessions through the Skype platform, which included intervention-addressed explicit instruction about the narrative structure and the use of discourse markers to improve cohesion in story retelling. Results: Significant changes were observed in the retelling of the story at microstructure and macrostructure levels: an increase of the Mean Length of Utterance (MLU), Types and Tokens, specific vocabulary, discourse markers and the recall of events. Conclusions: These results demonstrate the effectiveness of intervention in narrative skills through the oral retelling of a story with visual support via tele-practice.

## 1. Introduction

Developmental Language Disorder (DLD) (formerly known as Specific Language Impairment, SLI) [1] is understood to be a communication disorder that affects acquisition and development of language, which cannot be explained by the presence of other conditions, such as hearing loss, mental retardation, brain injury, psychological disorders, or poor socio-emotional adjustment [2]. It is considered that a child may be diagnosed with DLD when they show a delay of at least a year with respect to their chronological or mental age, excluding the following cases: (1) hearing below 25 dB, (2) emotional and behavioural problems, (3) minimal non-verbal CI under 85, (4) evidence of neurological deficits, and (5) severe phonological and/or articulatory problems [3]. The difficulties persist over time and problems may be observed not only on a linguistic level, but also in other areas, which leads to lower motor coordination [4], less symbolic play [5], as well as short-term visual memory [6] and deficits in auditory working memory [7].

The prevalence of this disorder is around 7% of the population without significant differences between sexes, although it is slightly more frequent among males [8].

DLD is considered to be a dynamic disorder, that is to say, in the same person, difficulties could be different over time, as well as diverse, since there exists a great variety of difficulties in those with DLD [3]. The first thing that is usually observed is a delay in the acquisition of vocabulary, but, as the child develops the areas of morphosyntax, phonology and pragmatics may also be affected [2].

According to Leonard and Deevy [9], the first difficulties in the lexical-semantic area may already be observed at a young age, since children with DLD show a delay in the acquisition of first words with respect to typical development and a lower number of different words, even though the acquired words are similar to those of children with typical development (objects, animals, etc.). Similarly, difficulties have been observed in access to lexical items, in the denomination and definition of verbs, nouns and adjectives, as well as problems in lexical organisation according to meaning [10].

With respect to pragmatics, it has been observed that it is an affected area that is independent from problems with morphosyntax and lexicon [2]. Within this field, difficulties which children with DLD experience in narrating stories stands out [11]. So, children with DLD produce poorer oral narratives on the micro-structural level (limited vocabulary, shorter and grammatically less complex sentences) and, on a macro-structural level, (fewer scenes, events, episodes and characters, fewer grammatical components, less organisation, coherence and general quality) compared with their peers with typical development [11,12,13,14,15,16,17,18,19]. Furthermore, difficulties may be observed in the production of complex syntactic structures, especially in sentences with markers of time, causality, purpose, comparatives and reversibles [20]. These problems are permanent, given that in adolescence limitations in the length of narratives, semantics and syntax continue to be seen [16,21]. This may have consequences on participation and access to the school curriculum [21], and in the creation and maintenance of social relations [22]. However, even though the narratives show the level of productivity and complexity of the language used, not enough attention has been paid to the narrative abilities of children with DLD [23]. Hence, intervention programs in narrative skills are also scarce [24,25,26].

This paper will concentrate on the oral narrative, which requires the integration and management of linguistic and cognitive abilities. When a person tells a story, they should choose those elements and details which are more important, they must keep the attention of the interlocutor, and they must be capable of making inferences from their knowledge of the world [27]. Narratives continue to develop over the years, including in adolescence, so that the number of narrative episodes increases as well as the ability to establish relations between them in a complex way, including emotions [28]. Similarly, it reflects the linguistic knowledge of the person, as well as their way of understanding the world and socio-cultural reality [29]. 

It has been observed that the oral retelling of a story offers an adequate model of narrative which includes the key components of a story, in contrast to that which occurs when first asked to read a story and then to tell it [12]. The retelling of stories could be a good strategy for facilitating the development of the narrative structure because it allows the child to reorganize the information as he tells it [30]. Similarly, the characteristic narrative of children differs according to the type of task with which they obtain the narrative (fiction or personal). In this sense, different types of tasks and methods (i.e., oral or visual sources such as a film, a single picture, or picture books such as containing the “Frog Story” [31] have been used to elicit oral narratives. Some studies suggest that the elicitation of oral narratives obtained from audio-visual material improves the linguistic characteristics of narratives [32].

On the other hand, the evaluation of elicited narratives for different tasks and methods is centred on measurements of microstructural linguistic features (i.e., vocabulary, morphology, Mean Length of Utterance (MLU), and syntax, primarily at the sentence level), and macrostructure elements of the narratives (i.e., content, organisation, and overall quality at the discourse level) [32,33,34]. Petersen [35] carried out an exhaustive literature survey and found only nine studies which evaluate narrative interventions of children between 3 and 21 years of age with language impairment. It was found that the narratives of preschool and school-age children with delayed and impaired language development increased both in level of microstructure and in macrostructure when derived from narrative interventions. This improvement in quality of the narrative in children could favour their capacity to participate in classroom activities and to benefit from these [36].

During the intervention sessions in this study, visual support was used, since this allows for observing language in more detail, for analysis, for revising it several times, for understanding and for maintaining it in long-term memory [37]. Furthermore, it reduces cognitive demands and so, it is supposed to be of great benefit to individuals with DLD [38,39] and it favours the linguistic characteristics of narratives [32].

Due to the current situation of the pandemic generated by COVID-19, speech therapy sessions for individuals with DLD and other pathologies have been altered. A strategy of American Speech-Language-Hearing Association (ASHA) [40] has been to include tele-practice in its methods, which is an alternative method that allows speech therapy evaluation and intervention to reach those clients who have communication disorders [41]. ASHA [40] defines telepractice as “the application of telecommunications technology to deliver professional services at a distance by linking clinician to patient, or clinician to clinician for assessment, intervention, and/or consultation”. 

The advantages that telepractice offers (accessibility, receiving the services of experts for client needs without having to travel, the attraction that digital devices can offer, effective results, the creation of businesses, the registration of data, and so on) has caused professionals to move from a certain reticence towards its use to a change in their perception [42] and a move towards embracing computer-based video-conferencing software to carry out clinical work at a distance [43].

As for the efficiency and satisfaction with the use of this type of practice, studies are scarce [44] and some are pilot studies [45,46]. Despite the scarcity of evidence, there are a few studies that show positive results in relation to this type of practice focused on both the patient and the caregivers [44,45,47,48,49,50,51,52,53], and it appears to be a very promising method in the case of children with difficulties in speaking and language [53].

Taking all this into account, the aim of this paper is the improvement of oral narrative abilities in a child of 9 years of age with DLD, through telepractice. Although there is evidence of the efficiency of narrative interventions in children with DLD on both a microstructural and macrostructural level in a situation of face-to-face speech therapy, it has not been observed whether these benefits can also be seen via telepractice.

The specific aims would be, on the one hand, to assess narrative competence of a child with DLD in relation to their ability to generate and orally retell a story based on an unpublished story and using linguistic measurements of microstructure and macrostructure. On the other hand, it aims to evaluate the effects of an explicit oral narrative intervention. The effects of the intervention on narrative microstructure and macrostructure were evaluated.

## 2. Materials and Methods

The research design was approved by the Ethics Committee for Research of the University of Oviedo. The study was developed in accordance with the code of ethics of the World Medical Association (Declaration of Helsinki) for experiments involving human subjects in research and the Spanish Law for Personal Data Protection (15/1999 and 3/2018) principles.

### 2.1. Participant

This is a case study of a Spanish boy of school age, 9 years and 2 months old, who will be called D. in order to remain anonymous. His first language is Spanish. Most of the time he lives with his mother, his older brother, and his stepfather. 

At the age of 3 he visited the ear, nose, and throat specialist due to recurring middle ear infections with hearing loss in that moment. At the age of 4 and 4 months he was observed to have language and/or communication difficulties, especially in phonological awareness. Because of this, the educational team at his school elaborated an Individual Work Plan and he began to see the specialist language, speech, and hearing teacher so as to work on phonological organisation, semantics, morphosyntax and pragmatics. This teacher concentrated, above all, on motor control, articulation and differentiation, and integration of phonemes. At 6 years of age, this student was evaluated and diagnosed by a speech therapist, who found that motor control was awkward and that he had difficulties both in expressive language (substitutions between fricatives, and difficulties with minimal pairs and poly-syllabics with minimal pairs, thus affecting syntactic structure, principally the use of verb tenses) and in language comprehension (he understood contexts and habitual instructions, but he had not acquired temporary organization). Given this clinical history, the speech therapist established a diagnosis of expressive DLD.

Considering all his difficulties at school, he was given pedagogical support and he also started to visit a speech therapist twice a week. Here he began to work on sequential memory in order to improve reading skills, as well as on concepts and time sequences, which are aspects related to the ability to narrate orally.

At the age of 7 years and 9 months, he was once again evaluated by a speech therapist who observed that oral language had improved, although he continued to have problems with written language. It was recommended that he continue to receive support from a speech therapist.

At the time of the study, he was in the 4th grade of primary school, which is aligned with his chronological age, and he continued to visit his speech therapist who focuses, above all, on learning support. He has also been given curricular adaptation. 

### 2.2. Previous Testing

Before the beginning of the intervention, D. was given a complete assessment. The results of that testing can be found in Table 1, Table 2 and Table 3.

The Peabody Picture Vocabulary Test (PPVT-III) [54] was administered at pre-intervention in order to evaluate possible deficits in vocabulary. His scores were found to be within the expected mean for his chronological age, with a score of 98 on linguistic IQ and a percentile of 45.

Table 1 shows the scaled scores obtained by the participant in the Test of Memory and Learning (TOMAL) [55]. The verbal memory index score is 98 while the index for non-verbal memory is 108. The participant obtained an expected score similar to his normative age group, except in the subtest Memory of Pairs, where his score was below mean.

The core language score in the test of Clinical Evaluation of Language Fundamentals (CELF-5) [56] shows that the participant’s score is found to be below the expected mean, obtaining the lowest score in the subtest Formulated Sentences test with three standard deviations below the expected mean. Table 2 shows the scaled scores from the CELF-5.

The results on the Illinois Test of Psycholinguistic Abilities (ITPA) [57] show that the participant´s psycholinguistic age from the sum of the direct scores is 10, somewhat above that for his chronological age. However, if we look at the scores obtained by the participant in each of the subtests (see Table 3), it can be observed that it shows a lower score with respect to his chronological age in Auditory Association, Sequential Auditory Memory and Sequential Visual Memory. So, the participant shows difficulties in relating concepts which are presented orally, in immediate memory of non-significant materials through the repetition of digits and in reproduction from memory of sequences of non-significant figures. Similarly, D. is found on the border of what is considered normal in two subtests: Visual Reception and Manual Expression. He shows greater difficulties in processing information received by oral input than by visuomotor input. 

### 2.3. Materials

#### 2.3.1. For the Assessment

The material used for the evaluation of the narrative level of the participant was the unpublished story titled *The Lost Backpack*. The story tells of a boy child, Fernando, who loses his backpack on the street while walking with his father. After a few hours the backpack is found by Alex, one of the characters in the story, who takes it home until the next day, when he decides to go to the Police Station to return it. When Alex is at the Police Station explaining that he found a backpack, Fernando appears with his parents. The child realizes that Alex has his backpack, so he approaches him with his parents, who prove it to him and, thus, they recover the lost backpack.

The narratives produced by the participant in the pre-test and in the post-test were recorded in MP3 format with an audio recorder incorporated into a Smartphone.

#### 2.3.2. For the Intervention

**Figure 1:**
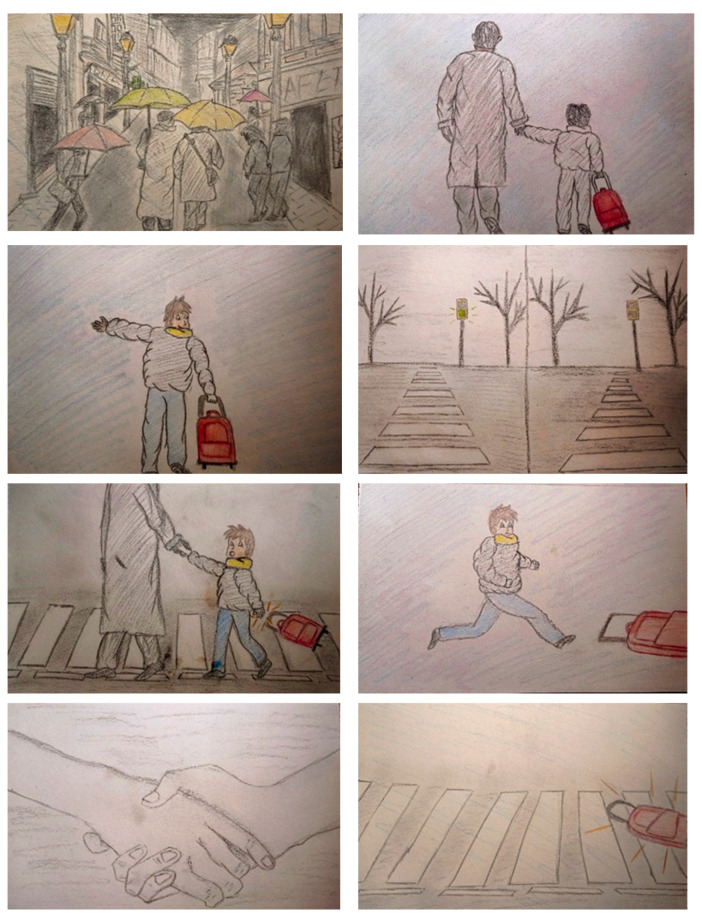
Pictures of events 1 to 4 of Scene 1.

The materials used for the intervention were 27 pictures which were designed by one of the researchers. Each drawing represented roughly one of the events of story. These pictures were used in the session interventions as visual support to facilitate comprehension of the story and to encourage narrative. Figure 1 shows the pictures used as visual support to narrate the first four events of Scene 1.

The JClic computer program [58], which is a free software, was used for designing proposed intervention activities. 

### 2.4. Procedure

The first meeting between the researchers, the family and the participant took place in a meeting on the Microsoft Teams MS platform, which is a platform which allows video meetings, and which also has other tools such as file storage and messaging by chat. In this first meeting the aims of the research and its duration were explained.

The initial evaluation process took place on three different days, since, for reasons of time, it was not possible to administer the four standardised tests in one day. The evaluation process was face-to-face in the participant’s home because some of the tests required motor responses which could not have been registered telematically and because, at the time, the incidence of COVID-19 was low, and the health authorities allowed it. The first evaluation session began with the application of the TOMAL test [55]. A number of rest periods were taken throughout the session because of the length of administration of the test. In the second evaluation session, the participant was given the main scales of the CELF-5 [56] which gave the core language score as well as the Peabody [54]. In the third evaluation session the ITPA [57] was administered. 

When the assessment of narrative abilities of the participant were about to begin, the health authorities of the region prohibited movement between cities. From then on, the pre-test and post-test evaluations of narratives skills, as well as the sessions of intervention, were carried out through video conferences on the Skype platform. Some of the characteristics of this platform allow screen-sharing with those who are interacting. So, it could be observed how the participant carried out the proposed activities, thus facilitating the interaction which is usually found in a face-to-face situation.

In the pre-test and post-test sessions to assess narrative abilities, the participant listened to the story *The Lost Backpack*. The participant was asked to pay special attention to the reading because later he would have to retell the story to the researcher. The story was then read to the participant, without visual support, and when it ended, D. was asked to orally narrate the story while being recorded on audio. The researcher used the verbal prompt “Tell me what you remember about the story”, so that D. began his narrative with no type of support or questions. Once his narrative ended, D. was asked a series of both inferential and literal questions related to the text in order to assess his degree of comprehension.

The intervention was divided into six weeks with two weekly sessions of approximately 40 min each day, except for the fourth and fifth weeks when there was only one session per week (a total of 10 sessions). Each week a different number of events were worked on, and these varied in complexity and length.

During the intervention sessions on Skype, the participant had to generate and retell one part of the story repeatedly with visual support and immediate scaffolding from one of the authors. The story was divided in four scenes (SCN), seven episodes (EPS) and 20 events (EVT), so that focus was placed on a specific number of events each session. A set of 27 pictures drawn by one of the authors was used as visual support during the intervention. Each picture represented one of the events in the “gold standard”. This is the complete version of the story built-up by the researchers, which served as the scheme for coding (see Table 4). The Rapid Pragmatic Evaluation Protocol (PREP-R) [59] was used for the coding of the macrostructure.

The intervention sessions began with a brief revision of the events worked on the week before, without scaffolding. Later, one of the authors presented new events using the set of pictures in a scripted way, through an activity specifically designed with the JClic program [58]. This program allows the creation of different text activities (word search, crossword puzzles, vignette order, display information…) where you can include images and audio by a native speaker. It also allows designing of the format, that is, changing the background, the sounds when the participant makes a hit or a mistake, vignette size, and font, as well as adding a time limit to carry out the activity; therefore, activities can be adapted to the participant’s interests and motivations. 

The intervention was organised around a sequence of actions that occur within each Scene, which is understood as the minimum unit of the narrative and which refers to a certain space and moment, highlighting the structure of the Events and the Episodes. In this way the structure of the narrative of the story is taught explicitly. The first and second sessions of intervention focussed on Scene 1, and specifically on Events 1, 2 and 3. The author showed the participant the set of pictures corresponding to the first Scene, one by one, presenting the characters, and testing explicit markers (MRK). Subsequently, D. was asked to narrate that specific scene with the support of the pictures, so his participation was required constantly. The third and fourth sessions were devoted to part of Scene 1 and the entirety of Scene 2 using the same methodology. In these sessions of intervention, the participant retold one part of Scene 1, having already retold the other part in the first and second sessions, and the entirety of Scene 2. The fifth and sixth sessions centred on part of Scenes 2 and 3. In order to do this, an activity designed with JClic [58] was used in which disordered pictures from the story appeared for the participant, so the objective was for D. to put the pictures in order while he told the story orally. The eighth session focused on events 14 and 15 of Scene 4. D. was also asked to narrate the whole story, comprising three scenes, and highlighting the sequential relationships within the general structure: initiating events, complicating actions, and high points. The ninth and tenth sessions were focussed on Scene 4 and on events 16 to 20 which is where the resolution of the story is found. In all the sessions, D. counted on visual support as well as questions, which he was asked by the researcher, for the progression of the story (“What happened next?”, “Who found the backpack?”, and so on). Table 5 shows the distribution of the events which were worked on by each session of intervention.

The activities programmed for each session, which included the events to be worked on, were sent by email a few hours before, so the participant could work on it by using his own computer. At the beginning of the session, he was asked to share his computer screen and, following this, he began to carry out the activities corresponding to that session.

Once the session was finished, a brief review was made of the events worked on, in chronological order, whether it was an activity created specifically for this (e.g., put pictures in order) or whether he was asked to orally narrate the sequence.

The intervention sessions were carried out in the living room of his home because this was the place where he had his computer, and the participant closed the door so that nobody would disturb the session. It was the participant himself who turned on the computer and joined the videoconference through his own account since he had enough technological ability to do so. Both the camera and the microphone were devices external to the computer itself and the connection was stable, so there were no interruptions or delays during the sessions. During the whole intervention session, the participant did not get up from his chair. He was continually accompanied by his mother or his stepfather, who helped him to maintain attention, if necessary. In general, the participant did the tasks without any complaints, although he was noticeably fatigued at the end of some sessions, given that these started after school hours and after extra-curricular activities.

### 2.5. Transcription and Coding

Both evaluation sessions were recorded with the aim of facilitating their later transcription and codification with the tools supplied by the CHILDES Project [60]. The narrative from both the pre-test and the post-test were coded in the CHAT format. The three major components of a CHAT transcript are the file headers, the main tier, and the dependent tiers. The header lines contain information about the date of the recording, the names of the participants, the age of the participants, the languages, the setting of the interaction, and so forth. The main lines give the basic transcription of what the speaker said. Each main tier line begins with an asterisk. After this, there is a three-letter speaker ID, a colon, and a tab. The dependent tiers are lines typed below the main line that contain codes, comments, events, and descriptions of interest to the researcher.

Transcription was conducted by the two trained researchers. In the first stage, the first author transcribed the pre-test and post-test recording, signalling all the unclear passages. In the second stage, the second author revised and resolved the final difficulties in order to achieve the highest agreement in the transcriptions. In the coding for microstructure and macrostructure, the first author coded the transcriptions from the pre-test and the post-test and, once that was done, the second author revised this coding. Cohen’s kappa was used to determine inter-rater reliability for coding of narrative structure (κ = 0.991; *p* < 0.001).

### 2.6. Measures

Coding for microstructure and macrostructure measures in the narratives was conducted in a different way. In the case of the microstructure, the total number of utterances (UTT), total number of clauses (CLA), and total number of words (tokens) (TOK) (microstructure productivity) were assessed, as well as the Mean Length of Utterance in words (MLUw), the total number of different words (Types) (TYP), and total number of discourse markers (MRK) (cohesive devices) (microstructure complexity). In the case of macrostructure, the total number of scenes, total number of episodes, and total number of events were assessed.

The coding of the macrostructure was based on the Rapid Pragmatic Evaluation Protocol (PREP-R) [59]. PREP-R allowed for coding of the narrative structure at three levels: (i) Scenes: basic or general level, corresponding to the locations or spaces in which the initiating event, complication, high point, and resolution of the story took place; (ii) Episodes: intermediate or integrated level, corresponding to sets of actions whose sequencing constitute the plot of the story; and (iii) Events: complex or detailed level, corresponding to the sequence of single actions making up the story. This story was divided into four scenes, eight episodes, and 20 events. From these, 16 words that were part of the Tokens of the story, and four discourse markers were selected with the aim of increasing cohesion in the final narrative. The narratives of the participant were compared with the complete version of the story, which served as the “gold standard” scheme for coding (see Table 4). 

To know if there had been a significant increase between the pre-test and the post-test, a percentage increase was calculated (Δ%) in the variable of the microstructure and macrostructure. The pre-test was taken as the reference value, and this value was subtracted from the post-test. The result was divided again by the reference value and, once the result was obtained, it was multiplied by 100 in order to find the previously mentioned percentage increase. These increases were found in order to compare progress in the microstructure of narrative productivity (UTT, CLA, TOK), and complexity (MLUw, TYP, MRK), as well as in the macrostructure of narrative productivity (SNC, EPS, EVT). Additionally, on the macrostructure level, we calculated the percentage of non-overlapping data (PND), that is a commonly used measure in case studies, which offers the advantage of comparing the data between the pre-test and post-test. To calculate the PND, we needed to know how many data points do not overlap and how many data points we have. In this case, the number of the non-overlapping was the difference between the scenes, episodes, and events recalled in the post-test and pre-test. Dividing the non-overlapping data points by all the data points calculated the percentage of non-overlapping data points. The result was multiplied by 100 for conversion into percentage form. Mastropieri et al. (1996) [61] have suggested that PND between 0 and 50 indicates an ineffective intervention, between 51 and 70 mildly effective, 71 and 90 moderately effective, and 91 and 100 indicates a highly effective intervention. 

## 3. Results

Table 6 shows the scores for productivity and complexity of narrative microstructure in the pre-test and in the post-test, as well as the percentage of improvement from one moment to the next. The results indicate that a significant increase was produced in the measures of microstructure between the pre-test and the post-test. After the intervention, the participant generated longer and more complex stories in terms of morphosyntactical and lexical measures. In the case of productivity in narrative microstructure, the three variables studied show a very high percentage increase, this being higher in Clauses (CLA), and in Tokens (TOK). In complexity of microstructure, the increases are more heterogeneous and extreme. In the MLUw, the percentage increase is lower, while in the MRK it is very high. The most-used discourse markers were ‘and’, ‘so’ and ‘then’.

Table 7 shows the scores for productivity of narrative macrostructure in the pre-test and in the post-test, as well as the percentage of improvement from one moment to the next. After intervention, the participant generated more complete stories at the integrated and detailed levels (episodes and events), and he included all the scenes. The increase is more significant in the case of events since he moves from a memory of 20% to 65% of the number of events into which the story was divided (20 events). In the case of episodes, the participant moves from a memory of 50% to 60% of the total for episodes. In both cases, the percentage increase is very high, greater than 10%. On the other hand, the percentage of non-overlapping in number of scenes is of 25%, of episodes is of 51% and events is of 45%.

## 4. Discussion

The aim of this case study was to improve the oral narrative abilities of a Spanish child, 9 years and 2 months of age, by the child retelling a story entitled *The Lost Backpack* which was elaborated by one of the authors using the strategy of working with tele-practice. The tasks of retelling the story and that of generating it constitute an adequate context for evaluation of narrative abilities because it is necessary to coherently organise information in order to retell the story. These tasks have been widely used in the evaluation of narrative abilities in individuals with DLD [12,13,14,15,16,17,18,19]. It has been observed that the production of narratives by those with DLD are shorter, syntactically simpler, with greater frequency of syntactic, semantic, and morphological errors, as well as with less cohesion [11]. However, as far as is known, there is no evidence for the efficiency of narrative interventions in children with DLD, on both a microstructural and macrostructural level, carried out telematically.

In the current pilot study, an oral narrative was elicited from an unpublished story in pre- and post-intervention sessions. These narratives were transcribed and coded for microstructures and macrostructure levels. On the microstructural level, measures of both productivity (utterances, clauses, and words) and complexity (MLUw, lexical diversity and use of discourse markers) were analysed. On the macrostructural level, only measures of productivity (the total number of scenes, episodes, and events) were analysed. 

Regarding the standardised tests, the participant obtained below average scores on the subtest for Memory of Pairs in the TOMAL. Additionally, he showed greater difficulties in the processing of information received in auditory input than in visuomotor input. Finally, the participant obtained scores below for his normative group in the CELF-5, with his lowest scores in Formulated Sentences. These results confirm the difficulties that the participant experiences when elaborating long, coherent sentences, and that he processes visual information better.

After the intervention, the participant showed significant improvement in all measures of narrative performance. The best results were observed on the microstructural level, with greater improvements in productivity (i.e., clauses and Tokens) than in complexity. On the macrostructural level, improvements were also seen in productivity, with the greatest advances being made in the number of remembered events. However, in terms of percentage of nonoverlapping data, the intervention was mildly effective in episodes, and was close to reaching this value for effective in events. In general terms, the participant created narratives with a greater number of, and longer, utterances (MLUw). Lexical diversity (Types) also increased. However, where the benefits were most striking was in the use of discourse markers. These had been explicitly taught during the intervention sessions. It seems that, by using the procedure described in this case study, the productivity and complexity microstructural level and productivity macrostructural level can be improved.

These improvements in language complexity and productivity allowed the participant to generate more complete oral narratives, thus increasing the number of remembered events. Similar to other case studies with language disorder participants [25,26] and a study with participants with Williams Syndrome [32], the improvements on a microstructural level, especially in the use of discourse markers, could be reflected in the narrative macrostructure when producing narratives with more detail. At the same time, the increase in microstructural measures on the level of productivity have favoured organisation and causal coherence after the intervention using visual materials. All of this could develop the production of temporal and causal sentences where those with DLD have difficulties [20]. Furthermore, the great number of exposures to the same words has facilitated learning [9] as well as organisation of lexicon [10] with respect to the generation of more elaborated oral narratives. As a result, the use of oral narratives in speech therapy interventions would generally improve the language of children with DLD [29]. 

The results obtained using telepractice as a working strategy agree with those found in other studies based on face-to-face intervention. Swanson et al. [36] carried out interventions in narrative abilities with a group of children with DLD by retelling different stories with visual support. Improvements in quality of narratives were observed, and these were quantified by taking into account the information related to scenes, events and the type of language used. Other studies have also obtained similar results in face-to-face intervention (see the review by Favot et al. [25] and Petersen [35]). However, in the study by Swanson et al. [36] no improvements were found in the Types, something which occurs in the present study. This is probably because a larger number of scenes, events and episodes was recalled.

On the other hand, it has been observed that the use of visual support facilitates the organisation of narrative structure [37]. In the initial evaluation, D’s narrative was incomplete, since he was required to tell the story after having listened to it, but without visual support. However, after the intervention, and after having used both visual and auditory stimuli, the narrative improved. From this, the use of visual support would seem to be an adequate strategy for intervention in these cases [38,39] because not only did the quality of the narrative increase but also the MLUw of the narrative, a very characteristic difficulty in children with DLD [13].

So, the retelling of stories with visual support facilitates the organisation of information as the story continues [30] and it seems to reduce cognitive demands [39]. This would allow children with DLD to concentrate more on linguistic aspects both in telematic and face-to-face interventions.

The efficiency of telepractice, both in language disorders as well as in other pathologies which affect communication, has not been studied in depth. It has centred mainly on the adaptation of the technology itself, on the comparison of results among children who have experienced recent intervention via telepractice as opposed to face-to-face, and on the attitudes of clients who participate in telepractice [50]. These studies have shown positive results after intervention via telepractice in the case of individuals with autism spectrum disorder (ASD), motor speech disorders, X Fragile Syndrome, and children with hearing impairment [47,48,49,62]. However, none have concentrated on individuals with DLD. The use of Information and communications technology (ICTs) and visual material via telepractice has been beneficial given that the participant in this study produced a more elaborate and complex oral narrative. It has been seen that intervention via telepractice could be associated with greater adherence to treatment in the case where the user shows autonomy and experience with the technical equipment [62]. The participant in this study has shown that he has a specific ability in the use of computers given that he was the one who turned on the computer, joined the videoconference through his account and shared his screen when carrying out the activities. These good computer skills could be another possible explanation for the results of the intervention, due to his involvement in the process.

In summary, this proposed intervention in oral narrative skills, through the use of a story and via telepractice, has not only improved coherence in narrative, but has also increased the number of Tokens, Types, MLU, as well as cohesion in narratives by increasing the number of discourse markers used by the participant after the intervention.

The main limitation of this study is the fact that the participant has been receiving the services of a speech therapist for approximately three years. Because of this, it is possible to considered that the results have been influenced by this factor. However, working with a story and a few specific words has allowed us to control for this variable to some extent. Furthermore, it may be added that the services of the speech therapist, which the participant currently receives, are aimed at learning support for schoolwork and not on intervention in linguistic aspects. Another limitation is that the case study and its results cannot be extrapolated to the entire population with DLD, even more so when dealing with such a heterogeneous and dynamic disorder. Moreover, a comparison of this type of intervention cannot be established between face-to-face intervention and intervention via telepractice because the pandemic did not allow for this possibility. Finally, despite the fact that complexity and productivity in microstructure and productivity in macrostructure increased after the intervention, this increase may have been more significant if a greater number of intervention sessions had been programmed. Therefore, this type of oral narrative intervention should be continued.

Future research needs to include a comparison condition in which the same methodology is applied to two groups of subjects with DDL, one by telepractice and another in a face-to-face situation, with observation for what leads to improvements in the microstructure and macrostructure level. Likewise, in this case study it would be necessary to study the impact of language treatment over longer periods of time. 

## Figures and Tables

**Table 1:** Scaled Scores for the Test of Memory and Learning (TOMAL).

	Tests	Scaled Scores
Verbal	Memory of Stories	11
	Selective Memory of Words	10
	Remembering Objects	10
	Direct Digits	15
	Memory of Pairs	5
	Direct Letters	9
	Inverse Digits	9
	Inverse Letters	9
Non-verbal	Memory of Faces	10
	Selective Visual Memory	9
	Abstract Visual Memory	12
	Sequential Visual Memory	16
	Memory of Places	10
	Manual Imitation	18
	Memory of Stories	11
	Memory of Faces	10
Delayed Memory	Selective Word Memory	12
	Selective Visual Memory	11

**Table 2:** Scaled Scores for CELF-5.

	Tests	Scaled Scores
Core Language Score	Word Classes	3
	Formulated Sentences	1
	Recalling Sentences	5
	Semantic Relationships	5

**Table 3:** Typical Scores obtained by the participant in the Illinois Test of Psycholinguistic Aptitudes (ITPA).

	Subtest	Typical Scores
Representational Level	Auditory Reception	35
	Visual Reception	45
	Auditory Association	24
	Visual Association	30
	Verbal Expression	48
	Manual Expression	35
Automatic-Sequential Level	Visual Sequential Memory	41
	Auditory Sequential Memory	34
	Visual Closure	30
	Grammatical Closure	35

**Table 4:** “Gold Standard” Scheme for Macrostructure Coding.

Scenes (SCN)	Episodes (EPS)	Events (EVT)	Pictures Correspondence
1. Street	1. A boy with a backpack is walking with his father.	1. It is raining and the streets are full of people. 2. Among the people, there is a boy who is walking with his father while carrying a big, red backpack.	1 2 & 3
	2. The boy and his father come to a pedestrian crossing and the boy drops the backpack.	3. The pedestrian lights are about to change to red, so they cross before this happens, but, the child lets go of the backpack. 4. The backpack is left on the ground. 5. Many people pass by but nobody picks up the backpack.	4, 5, 6 & 7 8 9
2. Doorway	3. Alex finds the backpack and takes it with him.	6. Alex walks by, stops and picks it up. 7. He takes shelter in the doorway to take a good look at the backpack and he sees a label with a name and an address. 8. He takes the backpack home.	10 11 & 12 13
3. Alex’s House	4. Alex takes the backpack and leaves the house.	9. After following his daily morning routine, he sits at his desk, turns on his computer and sees the backpack. 10. It is still wet and a bit dirty. 11. He gets up from his chair, puts on his coat and picks up the backpack.	14 & 15 16
4. The Police Station	5. Alex arrives at the police station to hand in the backpack.	12. He takes the backpack and goes to the police station. 13. He goes to one of the counters here he explains to a man what happened.	17 18 & 19
	6. The boy and his father come in.	14. Meanwhile, a boy, who is crying, enters the police station with his mother and father. 15. They go to the window next to Alex.	20 21
	7. The boy recognises his backpack, and it is returned to him.	16. The boy observes his backpack until he hears Alex’s explanation to the man behind the counter. 17. He goes up to Alex and tells him that it is his backpack. 18. His parents show it to him and the backpack is returned to him. 19. The boy opens the backpack and checks that his toys are still there.	22 23 24 25 & 26
	8. The boy and his parents say thank you to Alex and they say goodbye.	20. The four of them leave the police station and, in the doorway, they say thank you to Alex.	27

**Table 5:** Events worked on by each session.

Session	Events
First and Second	1, 2 & 3.
Third and Fourth	4, 5, 6, 7 & 8.
Fifth and Sixth	9, 10, 11, 12 & 13.
Seventh	Recall of 1 to 13. Introduction of 14 & 15.
Eighth	14 & 15.
Ninth and Tenth	16, 17, 18, 19 & 20.

**Table 6:** Microstructure of Narrative Productivity and Microstructure of Narrative Complexity.

		PRE	POST	Δ%
Productivity	UTT	6	31	416.66%
	CLA	4	46	1050%
	TOK	21	272	1195%
Complexity	MLUw	3.500	8.806	151.6%
	TYP	15	115	666.66%
	MRK	2	37	1750%

Note: UTT = Utterances; CLA = Clauses; TOK = Tokens; MLUw = Mean Length of Utterance in words; TYP = Number of different words; MRK = Discourse Markers.

**Table 7:** Macrostructure of Narrative Productivity.

	PRE	POST	Δ%
SCN	3	4	33.33%
EPS	4	8	100%
EVT	4	13	225%

Note: SCN = scenes; EPS = episodes; EVT = events.

## Data Availability

The data presented in this study are available on request from the corresponding author. The data are not publicly available due to ethical principles.

## References

[1] Bishop D.V., Snow P., Thompson P.A., Greenhalgh T., Catalise-2 Consortium (2017). Phase 2 of CATALISE: A multinational and multidisciplinary Delphi consensus study of problems with language development: Terminology. J. Child Psychol. Psychiatry.

[2] Leonard L.B. (1997). Children with Specific Language Impairment.

[3] Andreu L., Aguado G., Claustre M., Sanz-Torrent M. (2013). El Trastorno Específico del Lenguaje: Diagnóstico e Intervención.

[4] Johnston R.B., Stark R.E., Mellits E.D., Tallal P. (1981). Neurological status of language-impaired and normal children. Ann. Neurol..

[5] Udwin O., Yule W. (1983). Imaginative Play in Language Disordered Children. Int. J. Lang. Commun. Disord..

[6] Hick R., Botting N., Conti-Ramsden G. (2005). Cognitive abilities in children with specific language impairment: Consideration of visuo-spatial skills. Int. J. Lang. Commun. Disord..

[7] Montgomery J.W. (2002). Understanding the Language Difficulties of Children with Specific Language Impairments. Am. J. Speech-Lang. Pathol..

[8] Norbury C.F., Gooch D., Wray C., Baird G., Charman T., Simonoff E., Vamvakas G., Pickles A. (2016). The impact of nonverbal ability on prevalence and clinical presentation of language disorder: Evidence from a population study. J. Child Psychol. Psychiatry.

[9] Leonard L.B., Deevy P., Verhoeven L., van Balkom H. (2004). Lexical deficits in specific language impairment. Classification of Developmental Language Disorders: Theoretical Issues and Clinical Implications.

[10] Buiza J.J., Parra M.J.R., Adrián J.A. (2015). Trastorno Específico del Lenguaje: Marcadores psicolingüísticos en semántica y pragmática en niños españoles. An. Psicol..

[11] Norbury C.F., Bishop D.V.M. (2003). Narrative skills of children with communication impairments. Int. J. Lang. Commun. Disord..

[12] Boudreau D.M., Hedberg N.L. (1999). A Comparison of Early Literacy Skills in Children with Specific Language Impairment and Their Typically Developing Peers. Am. J. Speech-Lang. Pathol..

[13] Buiza J.J., Torres J.A., Sánchez M.G., Parra M.J.R. (2004). Evaluación de marcadores psicolingüísticos en el diagnóstico de niños con trastorno específico del lenguaje. Rev. Logop. Foniatr..

[14] Dodwell K., Bavin E.L. (2008). Children with specific language impairment: An investigation of their narratives and memory. Int. J. Lang. Commun. Disord..

[15] Duinmeijer I., De Jong J., Scheper A. (2012). Narrative abilities, memory and attention in children with a specific language impairment. Int. J. Lang. Commun. Disord..

[16] Fey M.E., Catts H.W., Proctor-Williams K., Tomblin J.B., Zhang X. (2004). Oral and written story composition skills of children with language impairment. J. Speech Lang. Hear. Res..

[17] Marini A., Tavano A., Fabbro F. (2008). Assessment of linguistic abilities in Italian children with Specific Language Impairment. Neuropsychologia.

[18] Purcell S.L., Liles B.Z. (1992). Cohesion repairs in the narratives of normal-language and language-disordered school-age children. J. Speech Lang. Hear. Res..

[19] Soodla P., Kikas E. (2010). Macrostructure in the narratives of Estonian children with typical development and language impairment. J. Speech Lang. Hear. Res..

[20] Buiza J.J., Rodríguez-Parra M.J., González-Sánchez M., Adrián J.A. (2016). Specific language impairment: Evaluation and detection of differential psycholinguistic markers in phonology and morphosyntax in Spanish-speaking children. Res. Dev. Disabil..

[21] Wetherell D., Botting N., Conti-Ramsden G. (2007). Narrative in adolescent specific language impairment (SLI): A comparison with peers across two different narrative genres. Int. J. Lang. Commun. Disord..

[22] Conti-Ramsden G., Botting N. (2004). Social difficulties and victimization in children with SLI at 11 years of age. J. Speech Lang. Hear. Res..

[23] Acosta V., Moreno A., Axpe Á. (2017). La detección e intervención en habilidades narrativas en niños con trastorno específico del lenguaje en contextos educativos (The detection and narrative skills intervention in children with specific language impairment in educational contexts). Educ. XX1 Rev. Fac. Educ..

[24] Spencer T.D., Slocum T.A. (2010). The effect of a narrative intervention on story retelling and personal story generation skills of preschoolers with risk factors and narrative language delays. J. Early Interv..

[25] Favot K., Carter M., Stephenson J. (2020). The effects of oral narrative intervention on the narratives of children with language disorder: A systematic literature review. J. Dev. Phys. Disabil..

[26] Klecan-Aker J.S. (1993). A treatment programme for improving story-telling ability: A case study. Child Lang. Teach. Ther..

[27] Andreu L., Sanz-Torrent M., Olmos J.G., MacWhinney B. (2011). Narrative comprehension and production in children with SLI: An eye movement study. Clin. Linguist. Phon..

[28] Liles B.Z. (1993). Narrative discourse in children with language disorders and children with normal language: A critical review of the literature. J. Speech Lang. Hear. Res..

[29] Acosta V., Moreno A., Axpe Á. (2012). Intervención logopédica sobre habilidades narrativas en niños con Trastorno Específico del Lenguaje. Infancia Aprendiz..

[30] Hoffman P.R., Norris J.A., Monjure J. (1990). Comparison of process targeting and whole language treatments for phonologically delayed preschool children. Lang. Speech Hear. Serv. Sch..

[31] Berman R.A., Slobin D.I. (1994). Relating Events in Narrative: A Crosslinguistic Developmental Study.

[32] Diez-Itza E., Martínez V., Pérez V., Fernández-Urquiza M. (2018). Explicit oral narrative intervention for students with Williams Syndrome. Front. Psychol..

[33] Heilmann J., Miller J.F., Nockerts A. (2010). Sensitivity of narrative organization measures using narrative retells produced by young school-age children. Lang. Test..

[34] Petersen D.B., Spencer T. (2012). The narrative language measures: Tools for language screening, progress monitoring, and intervention planning. Perspect. Lang. Learn. Educ..

[35] Petersen D.B. (2010). A Systematic review of narrative-based language intervention with children who have language impairment. Commun. Disord. Q..

[36] Swanson L.A., Fey M.E., Mills C.E., Hood L.S. (2005). Use of narrative-based language intervention with children who have specific language impairment. Am. J. Speech-Lang. Pathol..

[37] Monfort M. (2004). Intervención en niños con trastornos pragmáticos del lenguaje y la comunicación. Rev. Neurol..

[38] Gill C.B., Klecan-Aker J., Fredenburg K.A., Roberts T. (2003). Following directions: Rehearsal and visualization strategies for children with specific language impairment. Child Lang. Teach. Ther..

[39] Washington K.N., Warr-Leeper G.A. (2013). Visual support in intervention for preschoolers with specific language impairment. Top. Lang. Disord..

[40] Telepractice. https://www.asha.org/practice-portal/professional-issues/telepractice.

[41] Keck C.S., Doarn C. (2014). Telehealth technology applications in speech-language pathology. Telemed. e-Health.

[42] Ben-Aharon A. (2019). A Practical guide to establishing an online speech therapy private practice. Perspect. ASHA Spéc. Interes. Groups.

[43] Dudding C.C. (2008). Digital videoconferencing: A systematic review. Commun. Disord. Q..

[44] Wales D., Skinner L., Hayman M. (2017). The efficacy of telehealth-delivered speech and language intervention for primary school-age children: A systematic review. Int. J. Telerehabilit..

[45] Mashima P.A., Doarn C. (2008). Overview of Telehealth Activities in Speech-Language Pathology. Telemed. e-Health.

[46] Constantinescu G., Waite M., Dornan D., Rushbrooke E., Brown J., McGovern J., Ryan M., Hill A.J. (2014). A pilot study of telepractice delivery for teaching listening and spoken language to children with hearing loss. J. Telemed. Telecare.

[47] Boisvert M., Hall N., Andrianopoulos M., Chaclas J. (2012). The multi-faceted implementation of telepractice to service individuals with autism. Int. J. Telerehabilit..

[48] Bullard L., Abbeduto L. (2021). Responsive parenting as a target for telehealth language interventions in Fragile X Syndrome: Implications for scalability and best practices. Semin. Speech Lang..

[49] Isaki E., Farrell C.F. (2015). Provision of speech-language pathology telepractice services using Apple iPads. Telemed. e-Health..

[50] Law J., Dornstauder M., Charlton J., Gréaux M. (2021). Tele-practice for children and young people with communication disabilities: Employing the COM-B model to review the intervention literature and inform guidance for practitioners. Int. J. Lang. Commun. Disord..

[51] McCullough A. (2001). Viability and effectiveness of teletherapy for pre-school children with special needs. Int. J. Lang. Commun. Disord..

[52] Reynolds A.L., Vick J.L., Haak N.J. (2009). Telehealth applications in speech-language pathology: A modified narrative review. J. Telemed. Telecare..

[53] Waite M.C., Cahill L., Theodoras D.G., Busuttin S., Russell T. (2006). A pilot study of online assessment of childhood speech disorders. J. Telemed. Telecare..

[54] Dunn L.M., y Dunn L.M. (2010). PPVT-III: Test de Vocabulario en Imágenes (Peabody Picture Vocabulary Test. PPVT-III).

[55] Reynolds C.R., Bigler E.D. (2001). Test de Memoria y Aprendizaje: Manual (Test of Memory and Learning. TOMAL).

[56] Wiig E., Secord W., Semel E. (2018). CELF-5: Evaluación Clínica de los Fundamentos del Lenguaje (CELF-5: Clinical Evaluation of Language Fundamentals).

[57] Kirk S.A., McCarthy J.J., Kirk W.D. (2004). Test Illinois de Aptitudes Psicolingüísticas: Manual.

[58] JClic. https://clic.xtec.cat/legacy/es/jclic/.

[59] Fernández-Urquiza M., Miranda M., Martínez V., Diez-Itza E., Aguilar E., Adrover D., Bull L., López R. (2016). Pragmática textual de las narraciones en el síndrome de Down: Perfiles de coherencia y cohesión (Textual pragmatics of narratives in Down syndrome: Coherence and cohesion profiles). Proceedings of the VIIIth International Conference of Language Acquisition.

[60] MacWhinney B. (2000). The CHILDES Project: Tools for Analyzing Talk: Volume I: Transcription Format and Programs, Volume II: The Database.

[61] Mastropieri M.S., Scruggs T.E., Bakken J.P., Whedon C., Scruggs T.E., Mastropieri M.S. (1996). Reading comprehension: A synthesis of research in learning 707 disabilities. Advances in Learning and Behavioral Disabilities.

[62] Lowe R., O’Brian S., Onslow M. (2013). Review of telehealth stuttering management. Folia Phoniatr. Logop..

